# Morphological, cellular and molecular changes during postovulatory egg aging in mammals

**DOI:** 10.1186/s12929-015-0143-1

**Published:** 2015-05-22

**Authors:** Shilpa Prasad, Meenakshi Tiwari, Biplob Koch, Shail K. Chaube

**Affiliations:** Cell Physiology Laboratory, Biochemistry Unit, Department of Zoology, Banaras Hindu University, Varanasi, 221005 UP India; Genotoxicology and Cancer Biology Laboratory, Department of Zoology, Banaras Hindu University, Varanasi, 221005 UP India

**Keywords:** Postovulatory aging, Abortive SEA, Signal molecules, MPF, ART, Mammals

## Abstract

Postovulatory aging is associated with several morphological, cellular and molecular changes that deteriorate egg quality either by inducing abortive spontaneous egg activation (SEA) or by egg apoptosis. The reduced egg quality results in poor fertilization rate, embryo quality and reproductive outcome. Although postovulatory aging-induced abortive SEA has been reported in several mammalian species, the molecular mechanism(s) underlying this process remains to be elucidated. The postovulatory aging-induced morphological and cellular changes are characterized by partial cortical granules exocytosis, zona pellucida hardening, exit from metaphase-II (M-II)arrest and initiation of extrusion of second polar body in aged eggs. The molecular changes include reduction of adenosine 3',5'- cyclic monophosphate (cAMP) level, increase of reactive oxygen species (ROS) and thereby cytosolic free calcium (Ca^2+^) level. Increased levels of cAMP and/or ROS trigger accumulation of Thr-14/Tyr-15 phosphorylated cyclin-dependent kinase 1 (Cdk1) on **one hand and** degradation of cyclin B1 through ubiquitin-mediated proteolysis on the other hand to destabilize maturation promoting factor (MPF). The destabilized MPF triggers postovulatory aging-induced abortive SEA and limits various assisted reproductive technologies (ARTs) outcome in several mammalian species. **Use of** certain drugs that can either increase cAMP or reduce ROS level would prevent postovulatory aging-induced deterioration in egg quality so **that more** number of good quality eggs can be made available to improve ART outcome in mammals including human.

## Introduction

In mammals, freshly ovulated eggs are arrested at metaphase-II (M-II) stage of meiotic cell cycle and possess first polar body (PB-I) with normal morphology [1-3]. If fertilization does not occur within the window period soon after ovulation, unfertilized eggs remaining in the oviduct or under in vitro culture conditions, undergo time-dependent deterioration in quality by a process called postovulatory egg aging [4,5]. Postovulatory aging induces exit from M-II arrest and **initiation of second polar body (PB-II) extrusion** [2]. The chromosomes are scattered in the **cytoplasm and aged eggs are further** arrested at metaphase-III (M-III) like stage without forming pronuclei [2]. **The initiation of extrusion of PB-II occurs soon** after ovulation and large amount of cytoplasm **move** towards PB-II **area** but it never gets completely extruded. This atypical condition is called spontaneous egg activation (SEA) [2].

The SEA was reported in rat for the first time by Keefer and Schuetz in 1982 [6] and later by several **research** groups [2,5,7-11]. This pathological condition has also been observed in several mammalian species such as mice [12,13], porcine [14-16], bovine [17], hamster [18] and human **eggs** [19-21]. **The percentage of eggs undergoing SEA varies from species to species in mammals. Ross et al. (2006) observed that approximately 35 % to 85 % of ovulated eggs undergo SEA in different strains of rat. Studies from our laboratory suggest that the postovulatory egg aging results SEA in 90 % of ovulated eggs in vivo as well as in vitro** [2,3,8,9]**. In human, egg aging is one of the problems associated with ART failure** [4]**. Therefore, improvements of egg quality through methodological advances are in critical demand to prevent egg aging process during ART procedure** [4]**. Aged eggs limits the ART outcome, hence the establishment of method(s) to prevent egg aging could enhance progress in ART technologies and their outcome** [4]**. Based on our recent findings, we propose** that the postovulatory aging-induced abortive SEA could be due to changes in **the level** of signal molecules and their **effect** on maturation promoting factor **(MPF) because the high** level **of MPF heterodimer and cytostatic factors (CSF) activity** are required for maintenance of M-II arrest in freshly ovulated eggs [22,23]. **The postovulatory aging reduces egg quality by inducing apoptosis that finally affect reproductive outcome** [2,3,24-29].

## Review

### Morphological changes during postovulatory egg aging

**Fig. 1:**
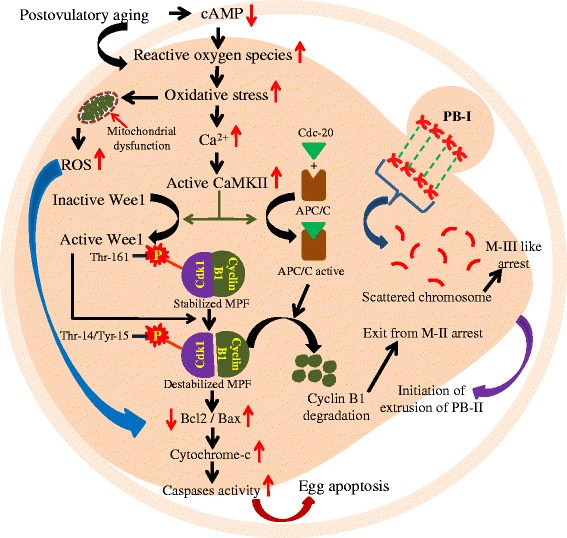
A schematic diagram showing the molecular changes associated with postovulatory aging-induced abortive SEA. Postovulatory egg aging reduces cAMP and/or induces generation of ROS that results in oxidative stress. The oxidative stress impairs mitochondrial membrane **potential and increases** cytosolic free Ca^2+^ level. The increased cytosolic free Ca^2+^ level results in the activation of Wee1 and APC/C. Wee1 **modulates** Cdk1 phosphorylation and **destabilizes** MPF heterodimer. The active APC/C triggers cyclin B1 degradation through ubiquitin-mediated proteolysis. **The destabilized** MPF triggers an exit from M-II arrest but chromosomes are scattered in the cytoplasm and pronuclei is not formed. The increased level of ROS and/or sustained destabilized MPF may trigger proapoptotic as well as apoptotic factors leading to apoptosis in aged eggs. **Increased cAMP level and decreased Ca** ^**2+**^ **and ROS levels using specific drugs could be beneficial to prevent postovulatory aging-induced deterioration of egg quality**

Normally egg activation is triggered by fertilizing spermatozoa and it is morphologically characterized by cortical granule exocytosis, pronuclei formation and complete extrusion of PB-II in mammal [2,12,30]. In the absence of fertilization, in several mammalian species, postovulatory aging induces SEA both in vivo as well as in vitro, which mimics the morphological features characteristics of egg activation [2,6,7,19,31,32]. **In rat,** postovulatory aging induces incomplete extrusion of PB-II without forming pronuclei and eggs are arrested at M-III like stage so called abortive SEA (**Fig.**1) [2]. The large amount of egg cytoplasm moves towards PB-II and **it is not cytoplasmic division, which** results partial extrusion of PB-II. The incomplete extrusion of PB-II generates a pathological condition because **these eggs** cannot be used **for assisted** reproductive technology (ART) program [2]. The postovulatory aging triggers degeneration of PB-I, increases perivitelline space (PVS) **and partial** cortical granule exocytosis [4]. **Due to energy depletion, postovulatory aging generates ROS and thereby egg apoptosis** [28,33]. The egg apoptosis has been morphologically characterized by shrinkage, membrane blebbing, cytoplasmic fragmentation, cytoplasmic granulation and degeneration [1,9-11,28,33-38].

### Cellular changes during postovulatory egg aging

Postovulatory aging causes tight aggregations of granulofibrillar material and zona pellucida hardening in eggs [4,12,39,40]. Thick microfilament domain underlying the plasma membrane is either disrupted or lost [41]. Number of lysosomes is increased and tubuli from smooth endoplasmic reticulum and small mitochondriacomplexes are aggregated in **aged** eggs [4,42]. Cortical granules are displaced and undergo **partial exocytosis** [4,12,39,40]. The mitochondria membrane potential is decreased, **which results in the swelling of matrix** [43]. Length of spindle is reduced **leading to** severe consequences for chromosome segregation. The centrosome structure, microtubule integrity and maintenance of chromosome at metaphase plate are lost [4,44,45]. As a **result,** pronuclei is not formed during postovulatory aging-induced abortive SEA (Fig. 1). Further, postovulatory aging triggers premature chromosome separation, chromosomal dispersion and decondensation, clumping of chromosome and chromatid separation in eggs [4,46,47] that **could** lead to epigenetic changes in offspring [23,48].

### Molecular changes during postovulatory egg aging

**The depletion of ATP reserve and thereby** adenosine 3',5'- cyclic monophosphate (cAMP) that leads to generation of reactive oxygen species (ROS) in aged eggs [33,37,38]. **The decrease of intracellular cAMP in aging eggs is one of the important signals that initiates an exit from M-II arrest** [2,49]. Few studies suggest **that reduced** cAMP level is associated with **an** increase **of hydrogen** peroxide (H_2_O_2_) in aged eggs cultured in vitro [50,51]. **The lack** of antioxidants and increased oxygen tension are other important factors that triggers the generation of ROS [13,23,52,53].

Increased oxidative stress due to generation of ROS **causes dysfunction** and shrinkage **of mitochondria** [23,38] **that reduces** mitochondrial membrane potential in aging eggs [53,54]. This is further supported by our observations that exogenous supplementation of dibutyryl cAMP (db-cAMP) or non-enzymatic antioxidant prevents postovulatory aging-induced abortive SEA [1-3]. Oxidative stress **induces** expression of proapoptotic factors (Bax and cytochrome c) and apoptotic factors **(caspase-3 and DNA fragmentation) and thereby apoptosis** in aged eggs cultured in vitro [9,55]. The increased level of H_2_O_2_ reduces Bcl2 expression [56], increases Bax expression [9,34,38], cytochrome c level, **caspases** activities [28,33,38,55] and DNA fragmentation in rat eggs cultured in vitro [9].

Postovulatory aging-induced oxidative stress can modulate RyR channels **of** endoplasmic reticulum and increase cytosolic free calcium Ca^2+^ level [3]. This is further strengthened by our **recent studies that ruthenium red, a** specific RyR channel **blocker reduces** cytosolic free Ca^2+^ level and inhibits postovulatory aging-induced abortive SEA [3]. Further, increased H_2_O_2_ level associates with **high** cytosolic free Ca^2+^ level during postovulatory-induced abortive SEA [1-3,10,36,38]. The increased intracellular Ca^2+^ activates CaM-dependent kinase-II (CaMKII) [3,57,58] **and** KN93, a specific CaMKII inhibitor prevents postovulatory aging-induced abortive SEA [57,58]. **On the other hand,** due to **high sustained level** of Ca^2+^ in the cytoplasm, Ca^2+^ enters in the mitochondria and triggers generation of ROS, mitochondrial DNA damage and apoptosis in aged eggs cultured in vitro (Fig. 1) [2,38,59,60].

The CaMK-II activates anaphase promoting complex/cyclosome (APC/C) by releasing endogenous meiotic inhibitor 2 (Emi2; a CSF) as well as Wee 1, a tyrosine kinase [61,62]. Previous study suggests **that increased** level of ROS can also stimulate tyrosine kinase [63]. The active Wee 1 destabilizes MPF by inducing phosphorylation of Thr-14/Tyr-15 of Cdk1 (a catalytic unit of MPF) and triggers **dissociation** of cyclin B1 (a regulatory subunit of MPF**) from MPF heterodimer** [61,62,64]. The active APC/C induces degradation of cyclin B1 through ubiquitin-mediated proteolysis [61]. The destabilized MPF finally triggers an exit from M-II **arrest and** thereby initiation of extrusion of PB-II in aged eggs [40]. The postovulatory aging-induced MPF destabilization can be prevented using several drugs that can elevate cAMP level or reduce ROS level [2,23,65,66]. Other drugs like demecolcine, nocodazole, cytochalasin (B and D) and Na^+^/Ca^2+^ exchanger of plasma membrane prevent postovulatory aging-induced abortive **SEA in rat oocytes** [67-69]. Although, postovulatory aging induces initiation of extrusion of PB-II but it never gets **completely** extruded and chromosomes remain scattered in egg cytoplasm without forming pronuclei [2]. The **reduced level of** destabilized MPF and ATP depletion in aged egg **result in increased** expression of proapoptotic factors [26,70,71]. **Overexpression** of proapoptotic factors activate upstream as well as downstream caspases [13,72] that deteriorates egg quality by inducing apoptosis [26,73-75]. Indeed, postovulatory aging-induced deterioration **of** egg quality could be one of the limiting factors for poor in vitro fertilization rate in several mammalian species including human.

## Conclusions

Postovulatory aging-induced abortive SEA is a pathological condition **in mammals that** limits ART outcome. Generation of ROS results in oxidative stress that **increases cytosolic free Ca**^**2+**^**level. Aged eggs are unable to sustained high level of Ca**^**2+**^**, which leads to MPF destabilization**. The destabilized MPF triggers exit from M-II arrest**, a** characteristic feature of abortive SEA (Fig. 1). **In aged eggs,** chromosomes are scattered in the egg cytoplasm and pronuclei is not formed. The increased oxidative stress and/or destabilized MPF deteriorate egg quality by inducing apoptosis. Although growing body of evidences suggest the possible players and pathways during postovulatory egg aging, further, studies are required to prevent aging process **so that the** good quality eggs **are made** available for various ART programs including somatic cell nuclear transfer during animal cloning.

## Abbreviations

ART: Assisted reproductive technology
APC/C: Anaphase promoting complex/cyclosome
cAMP: Adenosine 3',5'- cyclic monophosphate
Ca^2+^: Calcium
CaMKII: CaM-dependent kinase-II
Cdk1: Cyclin-dependent kinase 1
CSF: Cytostatic factor
db-cAMP: Dibutyryl cAMP
Emi2: Endogenous meiotic inhibitor 2
H_2_O_2_: Hydrogen peroxide
M-II: Metaphase-II
M-III: Metaphase-III
MPF: Maturation promoting factor
PB-I: Polar body-I
PB-II: polar body-II
PVS: perivitelline space
ROS: reactive oxygen species
SEA: spontaneous egg activation
